# Bibliometric and visualization analysis of stem cell therapy for meniscal regeneration from 2012 to 2022

**DOI:** 10.3389/fbioe.2023.1107209

**Published:** 2023-02-14

**Authors:** Zhen Yang, Zejun Fan, Du Wang, Hui Li, Zihao He, Dan Xing, Jianhao Lin

**Affiliations:** ^1^ Arthritis Clinical and Research Center, Peking University People’s Hospital, Beijing, China; ^2^ Arthritis Institute, Peking University, Beijing, China; ^3^ Department of Biomedical Engineering, School of Medicine, Tsinghua-Peking Center for Life Sciences, Tsinghua University, Beijing, China

**Keywords:** stem cell, meniscal regeneration, bibliometric, citespace, vosviewer

## Abstract

**Background:** Meniscus injuries, a common joint disease caused by long-term wear, trauma and inflammation, usually cause chronic dysfunction and pain in the joint. Current clinical surgeries mainly aim to remove the diseased tissue to alleviate patient suffering instead of helping with meniscus regeneration. As an emerging treatment, stem cell therapy has been verified to facilitate meniscus regeneration effectively. The purpose of this study is to investigate the publication conditions of stem cell therapy for meniscal regeneration and to visualize the research trends and frontiers.

**Methods:** Relevant publications relevant to stem cells for meniscal regeneration was retrieved SCI-Expanded of the Web of Science database from 2012 to 2022. Research trends in the field were analysed and visualized by CiteSpace and VOSviewer.

**Results:** A total of 354 publications were collected and analysed. The United States contributed the largest number of publications (118, 34.104%). Tokyo Medical Dental University has contributed the largest number of publications (34) among all full-time institutions. *Stem cell research therapy* has published the largest number of researches on stem cells for meniscal regeneration (17). SEKIYA. I contributed the majority of publications in this field (31), while Horie, M was the most frequently cited authors (166). #1 tissue engineering, #2 articular cartilage, #3 anterior cruciate ligament, #4 regenerative medicine, #5 scaffold are the chief keywords. This indicates that the current research hotspot has been transformed from basic surgical research to tissue engineering.

**Conclusion:** Stem cell therapy is a promising therapeutic method for meniscus regeneration. This is the first visualized and bibliometric study to thoroughly construct the development trends and knowledge structure in the research field of stem cell therapy for meniscal regeneration in the past 10 years. The results thoroughly summarize and visualize the research frontiers, which will shed light on the research direction of stem cell therapy for meniscal regeneration.

## 1 Introduction

The meniscus acts as a shock absorber between the shinbone and the thighbone, which is a C-shaped piece of tough, rubbery cartilage (Markes et al., 2020). In this sense, the meniscus plays a key role in normal knee movement and transmission of loading force, although the meniscus is smaller in size. Due to its high mechanical burden and continuous friction in the joint, it hurts easily and consequently becomes one of the most common knee injuries (Chambers and Chambers, 2019). On the one hand, while bearing weight on it, the meniscus can be torn if the knee is twisted suddenly due to inappropriate mechanical stimulation. On the other hand, excessive exercise and degeneration of articular cartilage usually cause meniscus wear, especially in aged individuals. Due to its avascular nature, the meniscus has a very weak ability to regenerate (Kurzweil et al., 2018). Thus, the chondrocytes are not able to proliferate fast enough and generate sufficient extracellular matrix to repair a defect (Kurzweil et al., 2018).

Researchers from all over the world are focusing on this field to find a way to alleviate the suffering and relieve symptoms of patients. For instance, some surgical accesses, already used in clinical treatment, can slightly alleviate patients’ suffering, including meniscectomy, which means removing the meniscus of the patients to reduce local chronic inflammatory reactions (Brown et al., 2021). Recent research results show that meniscectomy can only improve athletic ability in the beginning and has an adverse effect on the joint in several post-surgery years, although surgical technology provides symptomatic relief (Brown et al., 2021). When the knee joint moves, the friction between the femoral condyle and the tibial condyle increases. Under the same load, whether the meniscus is damaged or removed, the stress on the articular cartilage surface increases greatly. The decrease in stability promotes the subsequent formation of cartilage degeneration and osteoarthritis (Sun and Mauerhan, 2012).

As one type of stem cell, mesenchymal stem cells (MSCs) as the word implies, are named for their ability to differentiate into mesenchymal tissues (Yu et al., 2015). They have subpluripotent differentiation potential and can be induced into a variety of tissue cells in both natural and artificial environments. MSC therapy for meniscal regeneration is a relatively promising treatment, the main mechanism of which comes from its pluripotent differentiation potential (Caminal et al., 2014). Accordingly, when suffering from meniscal disease, the use of stem cell therapy may have corresponding therapeutic effects, especially for patients with mild articular meniscus wear and tears. In fact, mild patients are usually treated conservatively with no cure. With the development of regenerative medicine, MSC therapy has been tested for more than 10 years in the field of meniscus defects and meniscus regeneration (Joyce et al., 2010).

MSCs function mainly through two mechanisms of action (Hatsushika et al., 2013). On the one hand, stem cells directly repair damaged tissues through multidirectional differentiation potential, which means that stem cells are transformed into osteocytes and chondrocytes under specific induction conditions to repair bone and meniscus. On the other hand, stem cells activate the repair function of other cells, such as chondrocytes, by secreting various cytokines (Wang et al., 2017), such as transforming growth factor-β1 and insulin-like growth factor-1. These cytokines could inhibit the development of local inflammation and promote the self-repairing ability of local damaged tissues, thus accomplishing the purpose of meniscal regeneration.

In the last 10 years, many advances and achievements in stem cell-based therapy for meniscus repair have been made both clinically (Rinonapoli et al., 2021) and preclinically (Khalifeh Soltani et al., 2019). In addition to MSCs, many other cell types have also been utilized and further clinical applications require the effectiveness of clinical trials. In addition, in preclinical studies, different types of stem cells accompanied by fantastic biomaterials have been utilized to repair the meniscus, demonstrating a huge potential and a research hotspot. Despite the increasing results and interest in the topic of stem cell therapy for meniscal regeneration, the analysis of publication trends in this research area is still insufficient. Considering some researchers has summarized the specific advances (Le et al., 2020), there is a missing to comprehensively conclude the research development. Hence, it is necessary to explore, identify and analyse the trends in this field to help us predict and guide research directions.

As is widely acknowledged, the literature is the carrier of scientific progress. Bibliometric analysis uses mathematical and statistical methods to quantitatively and quantitatively analyse publications in medical databases, revealing the development history, research focus and future trends of a certain field. Among the literature, generous information was ignored. We are going to excavate useful information behind the data to help conclude and analyze the research development.

With advances in statistics science, bibliometric analysis has been utilized gradually, analysing current scientific research data and identifying developing trends (Wu et al., 2022a; IsmailSaqr, 2022). Furthermore, although a visualized network and comprehensive analysis, it is feasible to investigate the publication condition, predict research trends, and thus analyses research hotspots in specific fields (Zhang et al., 2022; Zhao et al., 2022). It is widely acknowledged that the combination of several bibliometric and mathematical tools, such as CiteSpace, the R package “bibliometrix” and VOSviewer, has also been widely used to visualize specific research literature analysis fields (Wu et al., 2022b; Zhang et al., 2022; Zhao et al., 2022). Accordingly, in this study, a new bibliometric statistics analysis with a visual network is performed to meet the unmet need and fill this knowledge gap. No related analysis of stem cell therapy for meniscal regeneration has been performed. This study comprehensively analysed the literature related to stem cell therapy for meniscal regeneration and performed visualization analysis over the last decade (2012–2022) to identify its research focus and even predict research hotspots. The study is significant because it is the first bibliometric analysis to scientifically and comprehensively analyze the researches related to stem cell therapy for meniscal regeneration and visualize the development trends in the past 10 years. Besides, the study would also benefit the audience a lot because it sheds light on the research direction of stem cell therapy for meniscal regeneration. On the one hand, current articles mainly focus on some specific therapy, which means, in fact, there is a lack of an all-round level bibliometric analysis of stem cell therapy for meniscus regeneration. On the other hand, researchers are in great need of such a summary, looking forward to understand the current status and hot spots in the field. An all-round level can help think clearly and avoid detours. Besides, a big data aggregation in the field can improve efficiency by reducing duplication of labor, sharing information resources, and improving researchers’ comprehensive capabilities. Furthermore, researchers could formulate more reasonable work goals and find more suitable scientific research directions when they read this study.

## 2 Materials and methods

### 2.1 Data source and search strategy

**FIGURE 1:**
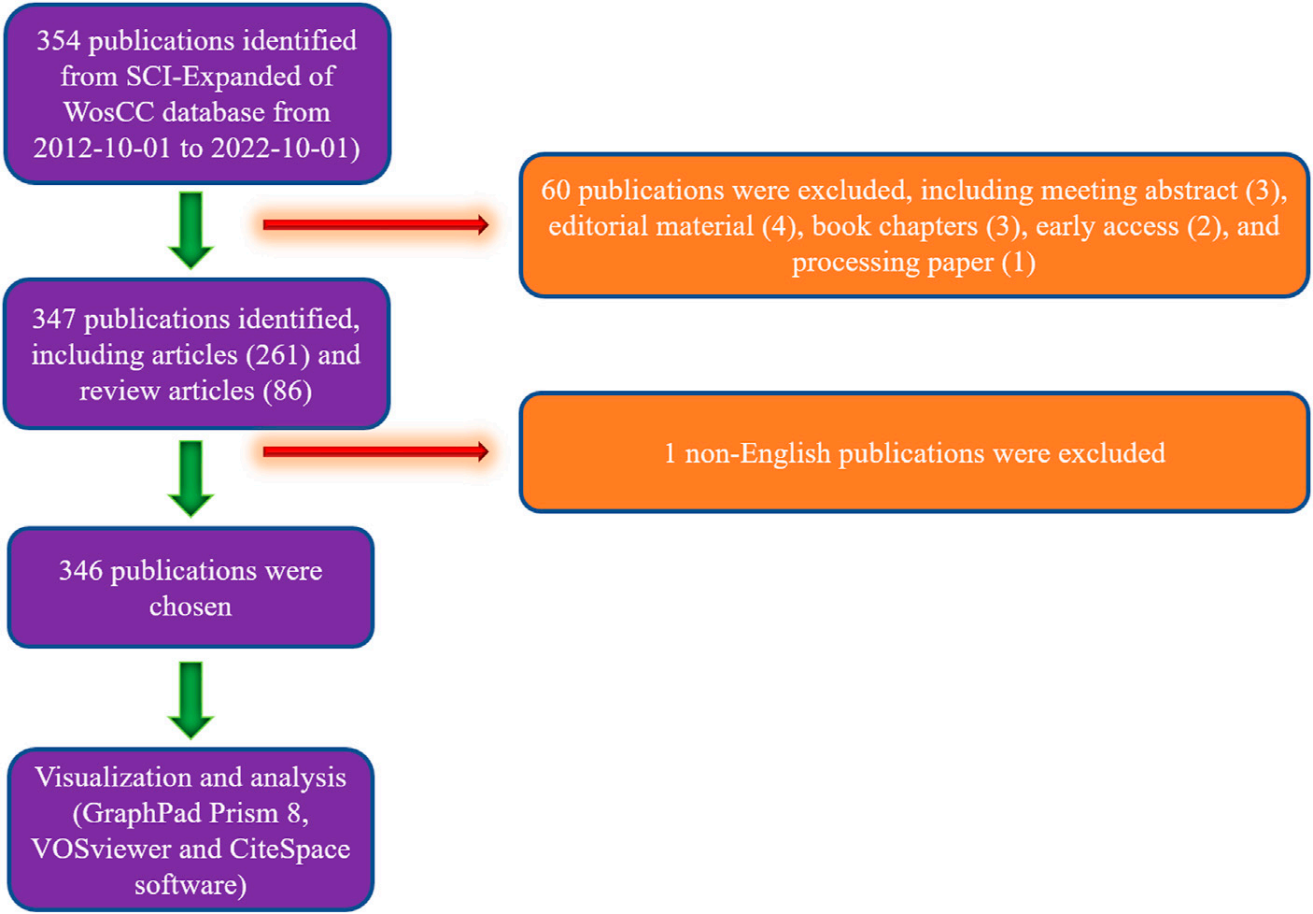
Flowchart depicting the article selection process.

As one of the most authoritative and comprehensive database platforms, the SCI-Expanded of Web of Science Core Collection (WoSCC) originates from Clarivate Analytics, which contains more than 12000 international academic journals (Wu et al., 2022b). Consequently, it was chosen to obtain global academic information for bibliometric analysis based on previous publications (Ekici et al., 2022). All the published literature was extracted from SCI-Expanded database, and the searching time was set from 1 October 2012 to 1 October 2022. The searching date is set to 3 November 2022, which means the update time of the dataset is determined. In present study, through the Advanced Search Section function, the searching terms were as follows (Figure 1): Theme = menisc* regeneration AND theme = stem cells AND publishing year = (2012.10.1–2022.10.1) AND Document types = (Article or Review) AND Language = (English). We indexed country/region to acquire further information of countries/regions in the SCI-Expanded database. The inclusion criteria are as follows: (1) Peer-reviewed publications mainly focused on the research field regarding stem cells for meniscal regeneration; (2) The document types must be Article and Review; (3) The publications should be written in English; (4) The publishing data must be between 2012 and 2022. The exclusion criteria were also as follows: (1) The themes of publications were not related to stem cells for meniscal regeneration; (2) Papers were news, meetings, abstract, briefings, etc.

In addition, all detailed data of the publications, including nationalities, name of journals, title, publishing year, author names, affiliations, abstract and keywords were saved in the format of download. txt files from the SCI-Expanded database and subsequently imported into Excel 2021. Coauthors (YZ and FZJ) searched and extracted all data from these studies independently. All disagreements were addressed by consulting with experts to reach the ultimate consensus. Finally, all data were cleaned and analysed individually by the coauthors and separately cleaned using GraphPad Prism 8 and Origin 2021.

### 2.2 Bibliometric analysis and visualization

The basic features of eligible studies were characterized by the intrinsic function of WoSCC.

Accordingly, the publishing number of studies and citations were analysed and visualized. We chose GraphPad Prism 8 and Origin 8 to perform the following bibliometric analysis. First, the year was taken as the *x*-axis and the number of documents published each year was set as *y*-axis to explore the trend of the number of documents issued. The relative research interest (RRI) was considered as the number of publications in a certain field by all field literatures per year (Shah et al., 2022). A combination of R software, including Python, NumPy, SciPy and Matplotlib, helped acquire the world map. Meanwhile, the time curve of publications has also been drawn. The H-index, which refers to a scholar who has published H papers and has been cited at least H times, was also calculated to measure the impact of scientific research (Hu et al., 2022).

To comprehensively visualize bibliometric networks of the publications, VOSviewer (Leiden University, Netherlands) software was used in this study. Deep down, bibliographic coupling, cocitation, and co-occurrence analysis were also performed and visualized in detail using VOSviewer. The relative parameter settings main about the minimum number of documents/citations/times. In details, 1) for bibliographic coupling analysis, the minimum number of documents of the Country, Journal, Author, and Institution was defied as more than 2; 2) for co-citation analysis, the minimum number of citations of the Author, Reference, and Journal was defined as more than10; 3) for co-authorship analysis, the minimum number of documents of the Country, Author, and Institution was defied as more than 2; 4) for co-occurrence analysis, the keyword was defined as the words used more than 2 times in titles/abstracts among all papers.

Moreover, CiteSpace (6.1. R2) served to construct a dual-map overlay for journals, visualize the diagram of country/regional collaboration, institutional collaboration and author collaboration, cluster analysis of co-cited keywords, and detection of references and keywords with intense citation bursts, which was developed by Professor Chen C. The parameters of CiteSpace were set as follows: link retaining factor (LRF = 3), look back years (LBY = 5), e for top N (e = 1), time span (2012–2022), years per slice (1), links (strength: Cosine, scope: Within slices), selection criteria (g-index: k = 25), and minimum duration (MD = 2 for keywords; MD = 5 for references).

## 3 Results

### 3.1 Overall performance of global literature

**FIGURE 2:**
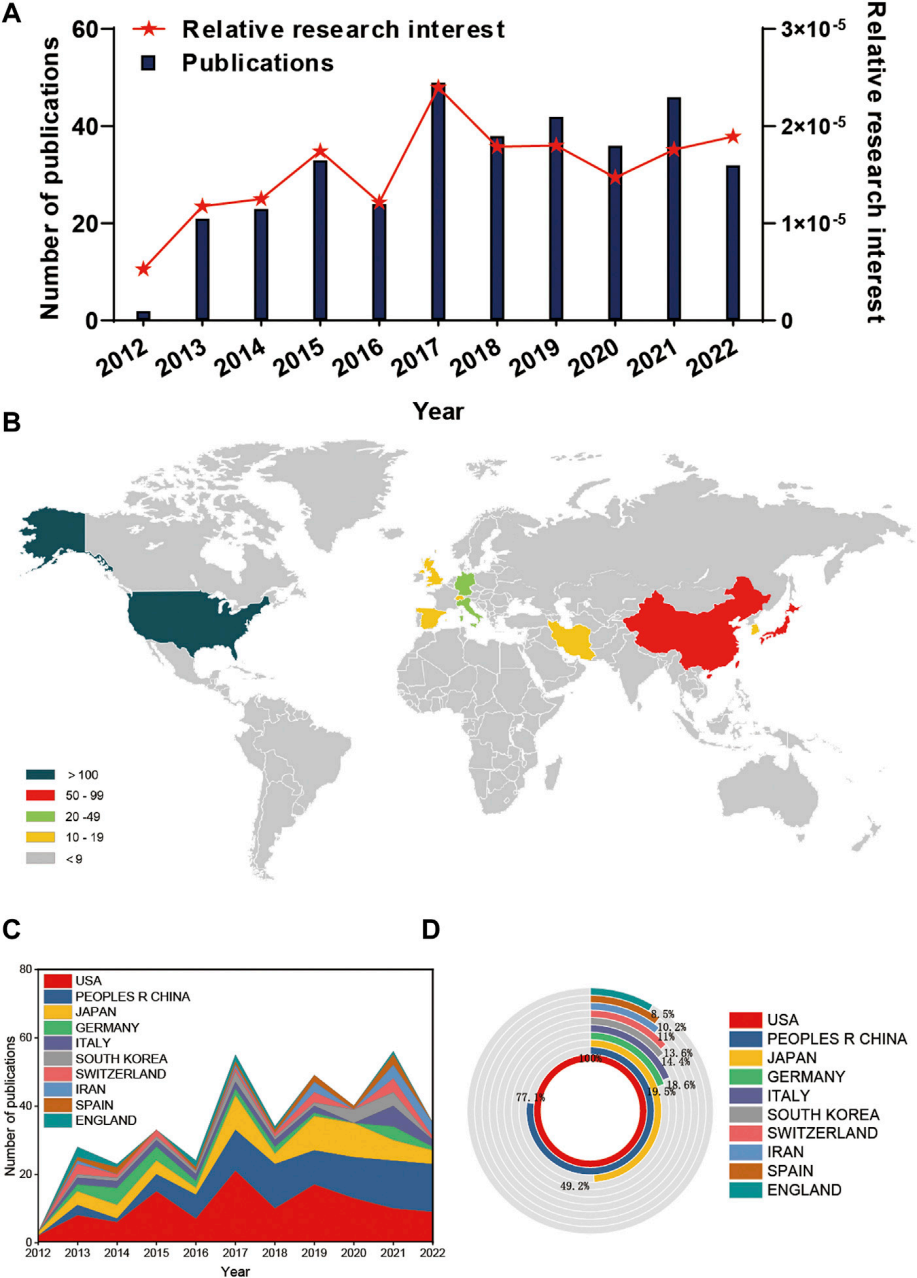
Global trends and countries/regions contributing to the research field regarding stem cells for meniscal regeneration from 2012 to 2022. **(A)** The annual number of publications related to stem cells for meniscal regeneration. **(B)** A world map depicting the distribution of stem cells for meniscal regeneration. The annual number **(C)** and total number **(D)** of publications in the top 10 most productive countries from 2012 to 2022.

Based on the search criteria, a total of 354 studies were collected and analyzed from 2012 to 2022. Second, 347 studies were identified, excluding the editorial material (4), meeting abstract (3), early access (2), correction book chapter (3), and processing paper (1). Consequently, 346 studies were identified, and 1 non-English study (Figure 1) was removed. As shown in Figure 2A, the trend of global literature experienced a steady increase almost year by year. The number of total global studies rose from 3 (2012) to 33 (2022). In the past decade, the year most studies were published was 2017 (Figure 2A). Additionally, there is ongoing increase in the research interest in this field over the past years in a not dissimilar way (Figure 2A).

Generally, 31 countries/regions have made contributions to the literature in this field according to VOSviewer. As suggested in Figures 2B, C, the United States contributed the most papers (117), followed by the four countries: China (91), Japan (58), Germany (23) and Italy (22). It is shown in Figure 2D that the United States proceeds much more in this the number of publications for the top 10 countries/regions. The number of publications of the second and third countries—China and Japan—are only equivalent to 77.1% and 49.2% of that of the United States, respectively.

From a temporal perspective, there is a slight decline in the annual number of publications in the United States and an increase in the number of publications in China in Figure 2C during 2019–2022. Overall, research on stem cells associated with meniscal regeneration has drawn increasing attention of global researchers and has arrived in a stage of rapid development.

### 3.2 Distribution of publications in countries

**FIGURE 3:**
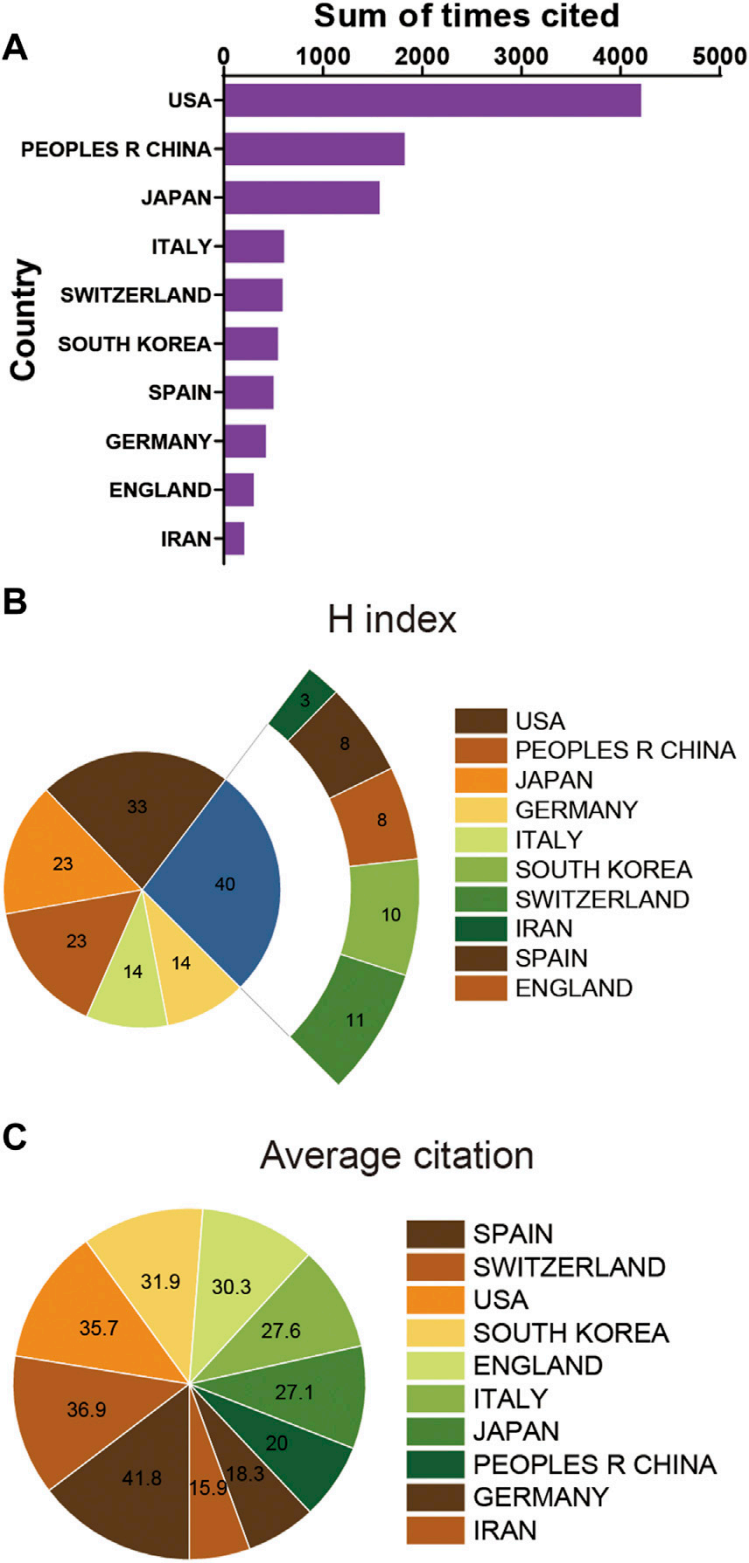
**(A)** The top 10 countries/regions of total citations related to stem cells for meniscal regeneration. **(B)** The top 10 countries/regions of the publication H-index related to stem cells for meniscal regeneration. **(C)** The top 10 countries/regions of the average citations per publication related to stem cells for meniscal regeneration.

As seen from Figure 3A and Table 1, the publications with the highest total citation frequencies were form the United States (4184). China ranked second concerning total citation frequencies (1822), followed by Japan (1569), Italy (608) and Switzerland (591). Additionally, the United States (33) played a dominant role in this field in the relative publications of the H-index, followed by China (23), Japan (23), Germany (14) and Italy (14) (Figure 3B). Interestingly, when it comes to average citation frequency, publications from Spain possessed the highest average citation frequencies (41.8). Switzerland ranked in the second position in the aspect of average citation frequency (36.9) prior to the United States (36.9), South Korea (31.9) and England (30.3) (Figure 3C).

**TABLE 1:** The top 10 most productive countries/regions related to stem cells for meniscal regeneration.

Rank	Country/region	Article counts	Percentage
1	United States	118	34.10
2	Peoples R China	91	26.30
3	Japan	58	16.76
4	Germany	23	6.64
5	Italy	22	6.36
6	South Korea	17	4.91
7	Switzerland	16	4.62
8	Iran	13	3.76
9	Spain	12	3.47
10	England	10	2.89

### 3.3 Analysis of country/regional collaboration

**FIGURE 4:**
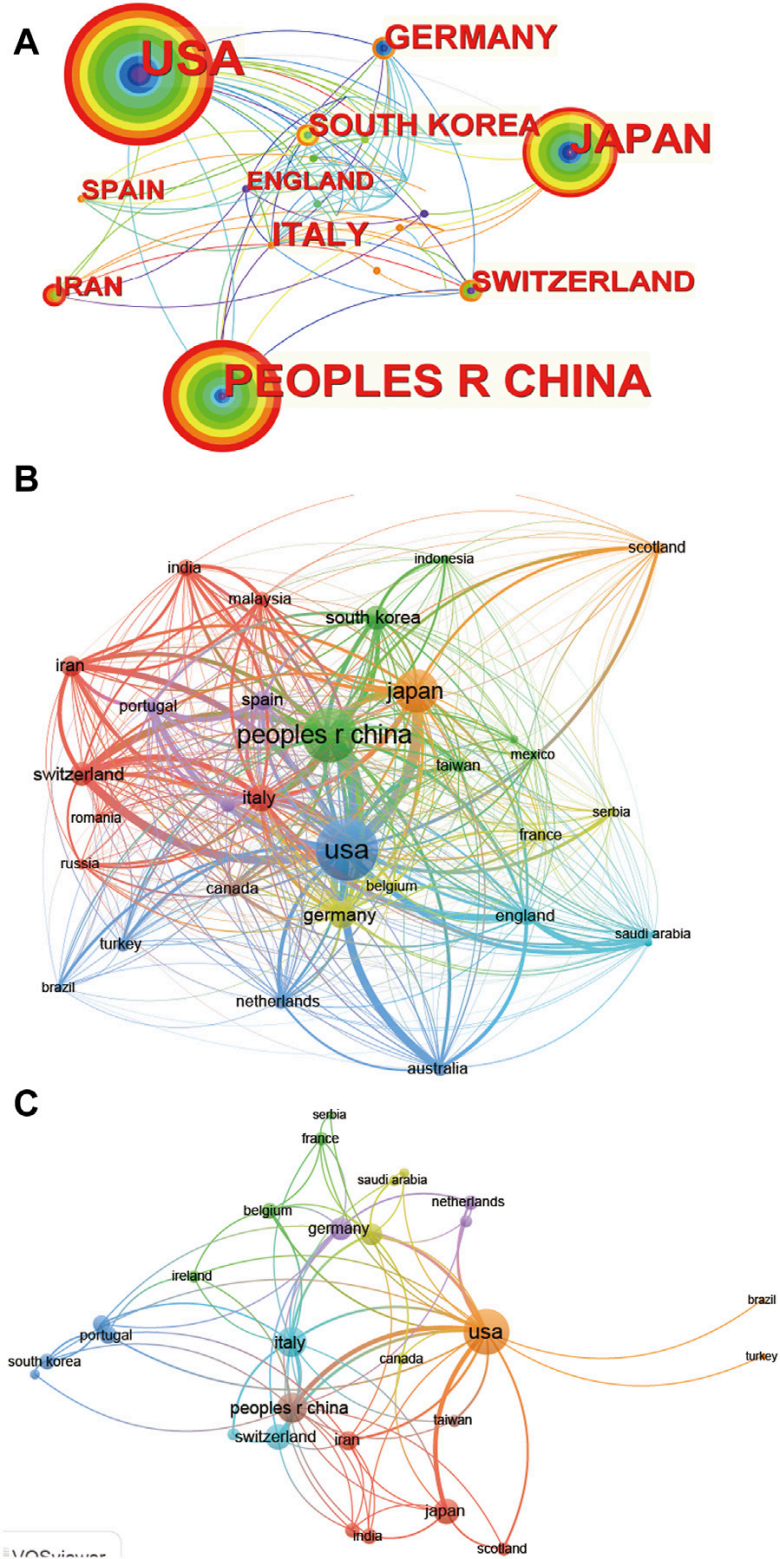
Mapping of countries/regions associated with stem cells for meniscal regeneration. Country/regional collaboration analysis derived based on CiteSpace **(A)** and Vosviewer **(B)**. **(C)** The authorship-country collaboration analysis *via* Vosviewer. The nodes represent countries/regions, and the lines connect them. The number of publications grows proportionally to the size of the nodes. The lines between the nodes represent the cooperation relationship, and the thickness of the connecting lines represents the strength of their cooperation; the closer the cooperation is, the thicker the connecting lines. The nodes with the outermost orange circles have higher centrality.

The global collaboration network analysis was also carried out; Figure 4A shows that the United States exhibited the highest output area and worked closely with other countries using CiteSpace, which means it had the strongest international collaboration. In detail, the size of the circle represents the collaboration strength, while the color represents the distribution of collaboration time from 2012 to 2022. From Figure 4A, the United States, China and Japan have maintained a steady collaboration strength in the last decade. However, Germany experiences shrinking international collaboration with other countries.

Additionally, the collaboration of the 31 countries is visualized in Figures 4B, C. Figures 4B, C shows that the United States (link strength 5255.64), Japan (link strength 3377.95) and China (link strength 1722.27) have the most frequent international collaborations. In addition, the majority of international partners of the United States are Australia, Japan and Switzerland. The transformation of collaboration with time can also be acquired from the color of the circle of country and the link between different countries, which did not represent obvious changes for most countries.

### 3.4 Analysis of institutions

**FIGURE 5:**
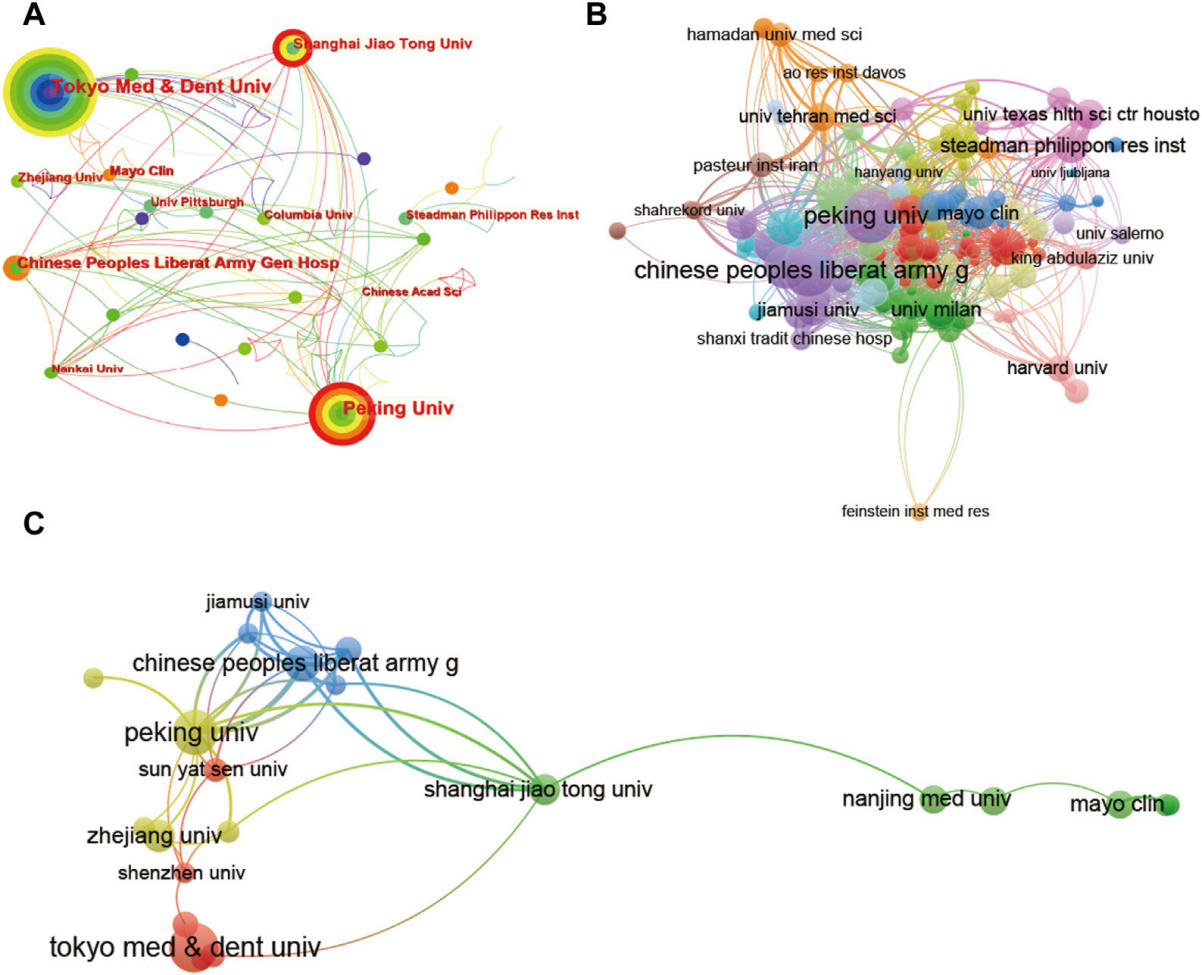
Mapping of institutions associated with stem cells for meniscal regeneration. Institutional collaboration analysis based on CiteSpace **(A)** and Vosviewer **(B)**. **(C)** The authorship-institution collaboration analysis *via* Vosviewer. The nodes represent countries/regions or institutions, and the lines connect them. The number of publications grows proportionally to the size of the nodes. The lines between the nodes represent the cooperation relationship, and the thickness of the connecting lines represents the strength of their cooperation; the closer the cooperation is, the thicker the connecting lines. The nodes with the outermost red circles have higher centrality.

Regarding publication ranking, the top 10 contributing institutions are listed in Table 2 and visualized in Figures 5A, B. Based on CiteSpace in Figures 5A, B, in terms of international collaboration between institutions, the first institution was Tokyo Medical and Dental University, which also possessed a high yield of publication. Interestingly, the time of the main collaboration lasted from 2012 to 2017, which means that there is currently a decrease in collaboration. In contrast, the two Chinese institutions, Peking University and Shanghai Jiao Tong University, display an intense collaboration strength from 2017 to the present, ranking second and third, respectively. Based on Vosviewer in Figure 5B, the impact of each research is estimated by the citations, considering the citations in the 161 institutions. In that sense, the first institution is Peking University (link strength 1646.14), followed by People’s Liberation Army General Hospital (link strength 1567.14) and Nankai University (link strength 1041.34). Especially concerning Nankai University, the research number is not very high, yet the research impact is relatively strong. Figure 5C demonstrated the network diagram of collaboration between institutions. As suggested, there is a close cooperative relationship between institutions in East Asia, such as Shanghai Jiao Tong University, Tokyo Medical and Dental University, People’s Liberation Army General Hospital and Peking University.

**TABLE 2:** The top 10 institutions published literature related to stem cells for meniscal regeneration.

Rank	Institution	Article counts	Percentage	Country	Total citations	Average citation
1	Tokyo Medical Dental University Tmdu	34	9.83	Japan	1231	34.19
2	Peking University	29	8.38	China	508	17.52
3	Chinese People S Liberation Army General Hospital	15	4.36	China	272	18.13
4	Pennsylvania Commonwealth System of Higher Education Pcshe	13	3.76	United States	593	45.62
5	Shanghai Jiao Tong University	13	3.76	China	319	24.54
6	Zhejiang University	13	3.76	China	413	31.77
7	Harvard University	11	3.18	United States	319	26.58
8	University of Regensburg	11	3.18	Germany	253	23.00
9	Mayo Clinic	10	2.89	United States	244	24.40
10	Nanjing Medical University	10	2.89	China	142	14.20

### 3.5 Analysis of authors

In general, a total of 326 authors in this field that have more than 2 articles are considered and calculated by Vosviewer and CiteSpace. The relatedness of the items depending on the numbers they together co-operated in one study was calculated in the collaboration analysis.

**FIGURE 6:**
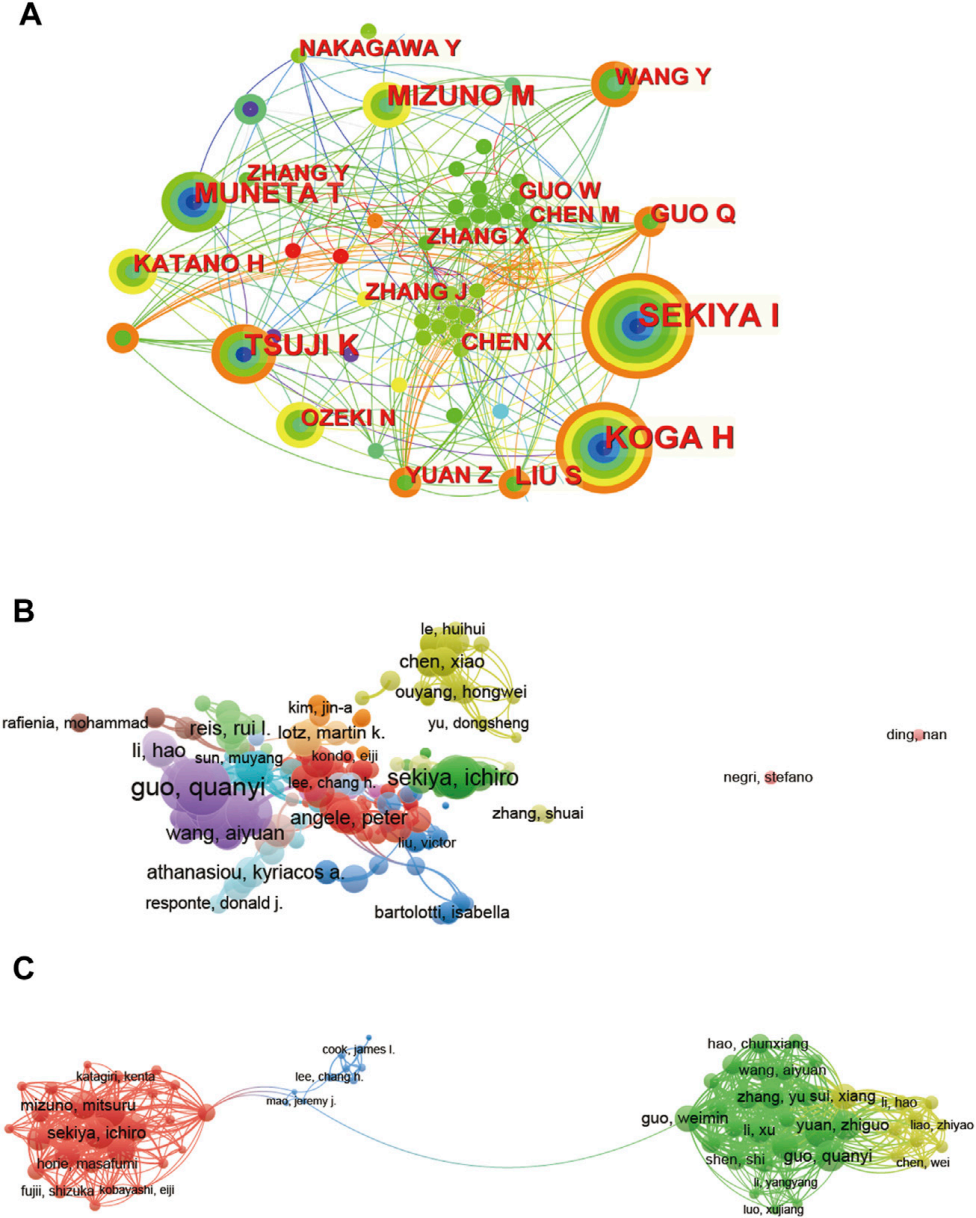
CiteSpace and Vosviewer network visualizations of author collaboration analysis regarding stem cells for meniscal regeneration. Author collaboration analysed by CiteSpace **(A)** and Vosviewer **(B)**. **(C)** Network visualization diagram of authorship-author analysis based on Vosviewer. Author collaboration authors are indicated by the node. The collaboration relationship is indicated by the line connecting the nodes. The node area grows as the number of collaborations increases. The colors from green to orange represent different years from 2012 to 2022.

All required authors were analysed using CiteSpace (Figure 6A) and Vosviewer (Figure 6B). Based on CiteSpace (Figure 6A), the top 5 authors with the largest total link and circle strength were as follows: SEKIYA I, KOGA H, MUNETA T, TSUJI K, MIZUNO M. In detail, Sekiya published 27 articles with 748 citations, which proves its contribution and leading role in the field from these two aspects. It is also suggested that collaborations between American and Japanese researchers are much more frequent and closer. Based on Vosviewer (Figure 6B), the visualization of author collaboration shows that there exist some collaboration circles of researchers, the central authors of which are Guo, Sekiya, Chen and Angele. In Figure 6C, a clear transition of collaboration with time from 2012 to 2022 has been displayed, predicting a possible transfer of academic center. According to Figure 6C, it seems that during 2012–2022, a hot collaboration will transfer from Guo to Mao and then to Sekiya.

### 3.6 Citation and Co-Citation analysis

**FIGURE 7:**
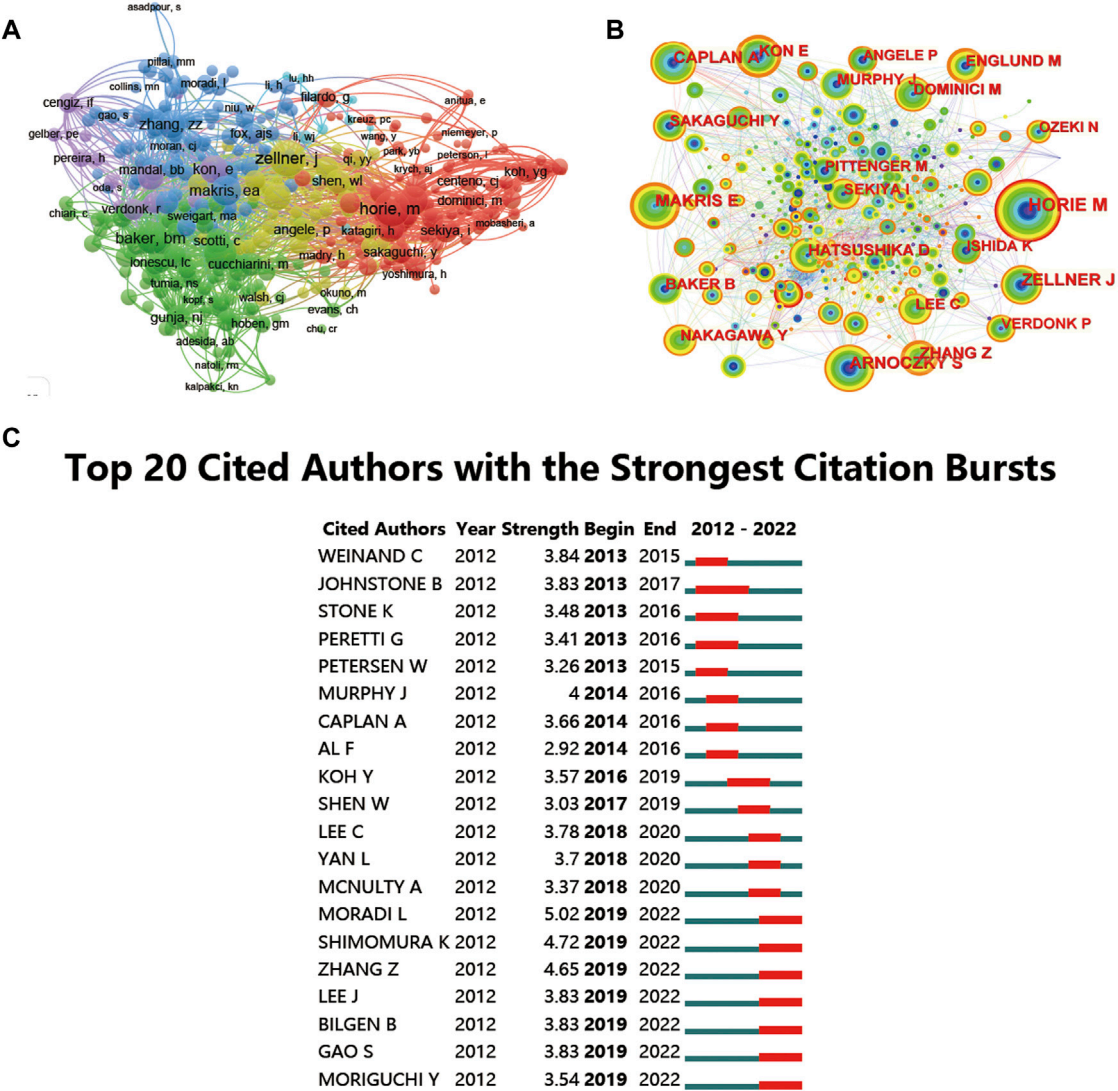
CiteSpace network visualization of cocitation author analysis regarding stem cells for meniscus regeneration. Author cocitation analysed by Vosviewer **(A)** and CiteSpace **(B)**. **(C)** Top 25 references with the strongest citation bursts of publications related to stem cells for meniscal regeneration. Author cocitations are indicated by the node. The cocitation relationship is indicated by the line connecting the nodes. The node area grows as the number of cocitations increases. The colors from green to orange represent different years from 2012 to 2022.

In terms of citation and cocitation analysis, a total of 367 researches in this field have been collected. These publications collected are more than 10 citations (Figure 7A). The leading 5 most cited documents are listed in Table 3. There were 301 citations for “Treatment of Knee Osteoarthritis with Autologous Mesenchymal Stem Cells: A Pilot Study”, followed by “Adult Human Mesenchymal Stem Cells Delivered *via* Intraarticular Injection to the Knee Following Partial Medial Meniscectomy a Randomized, Double-Blind, Controlled Study”, with 255 citations. The third-dominant article with the largest number of citations was “Poly (lactic acid)-based biomaterials for orthopaedic regenerative engineering”, with 223 citations. Cocitation means that an article is cited by two different articles but not necessarily linked. Moreover, cocited authors were analysed by CiteSpace (Figure 7B) to show the top 5 most influential studies.

**TABLE 3:** The top 5 documents with the most citations in the field of stem cells for meniscal regeneration.

Rank	Title	First author	Journal	IF	Publication year	Total citations
1	Treatment of Knee Osteoarthritis with Autologous Mesenchymal Stem Cells: A Pilot Study	Orozco, L	Transplantation	5.385	2013	301
2	Adult Human Mesenchymal Stem Cells Delivered *via* Intra-Articular Injection to the Knee Following Partial Medial Meniscectomy A Randomized, Double-Blind, Controlled Study	Vangsness, CT	Journal of Bone and Joint Surgery-American Volume	6.558	2014	255
3	Poly (lactic acid)-based biomaterials for orthopaedic regenerative engineering	Narayanan, G	Advanced Drug Delivery Reviews	17.873	2016	223
4	Surgical and tissue engineering strategies for articular cartilage and meniscus repair	Kwon, H	Nature Reviews Rheumatology	32.286	2019	201
5	Secreted trophic factors of mesenchymal stem cells support neurovascular and musculoskeletal therapies	Hofer, HR	Stem Cell Research & Therapy	8.079	2016	201

What can’t be ignored is citation burst, which is a valuable indicator in a particular domain in a period, reflecting the references of interest to researchers. In our analysis, the top strongest citation bursts were characterized and identified. In details, a total of 20 publications have been summarized by CiteSpace and are presented in Figure 7C. The study by Weinand C maintains the strongest citation burst with a strength of 3.84, lasting from 2013 to 2015.

### 3.7 Analysis of research areas and journals

**FIGURE 8:**
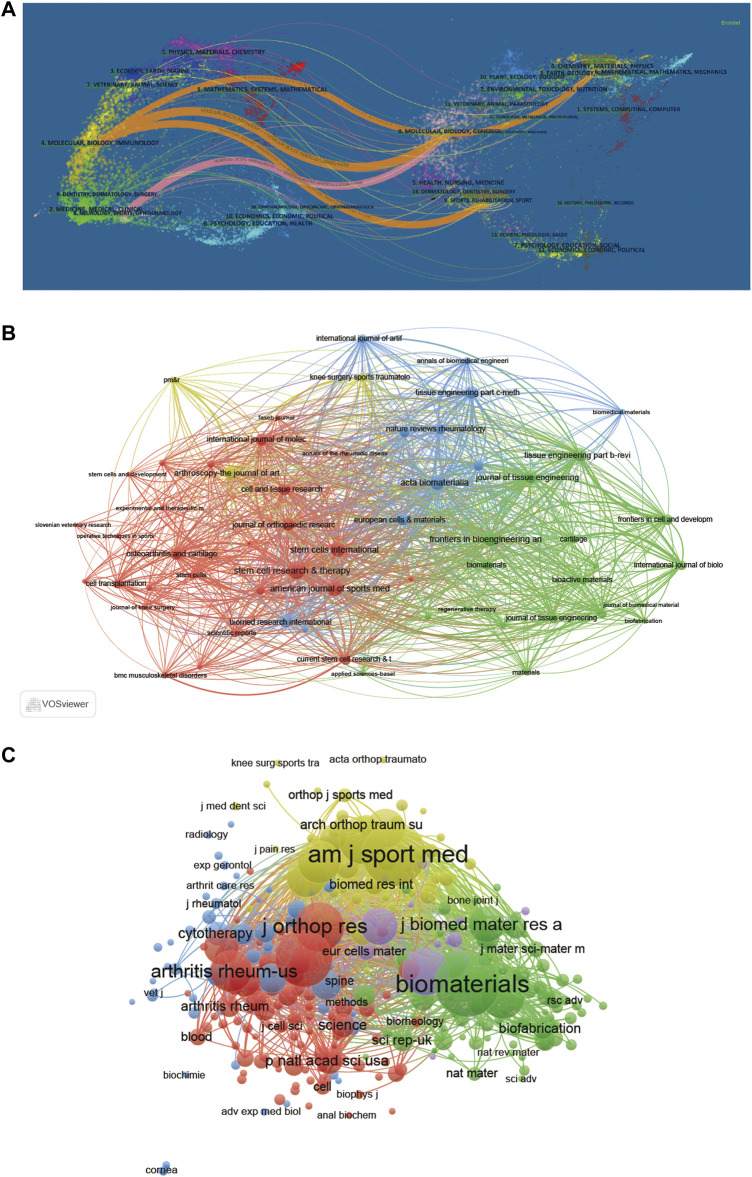
Articles published in different journals on stem cells for meniscal regeneration. **(A)** The dual-map overlay of journals related to stem cells for meniscal regeneration. **(B)** Bibliographic analysis of journals based on Vosviewer. **(C)** Network map of journals that were cocited in more than 50 citations based on Vosviewer.

**FIGURE 9:**
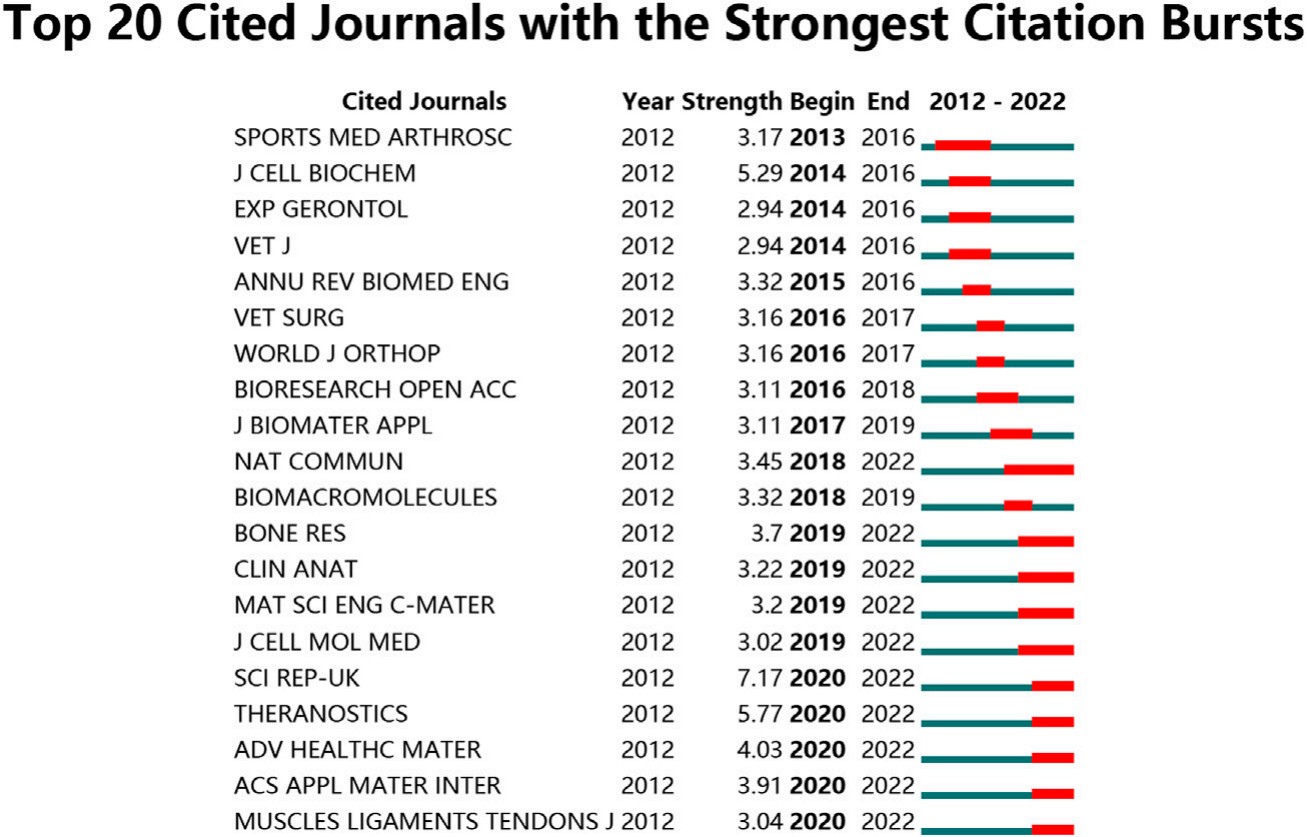
Top 20 cited journals with the strongest citation bursts of publications related to stem cells for meniscal regeneration.

The 10 most productive journals involved in this study have been displayed in Table 4. For visualization, the dual-map overlay of journals related to stem cells for meniscal regeneration is plotted after analysis in Figures 8A, B, 9. The journal *Stem Cells Research Therapy* published the most, with 17 publications. There were 15 publications in *American Journal of Sports Medicine*, 13 publications in *Journal of Orthopaedic Research*, 12 publications in *Acta Biomaterialia* and 9 articles in *Osteoarthritis and Cartilage*. Concerning the analysis of journals of cocitation using VOSviewer, the collection standard is that the journal with a number of citations less than 10 will be ignored. As visualized in Figure 8C, 300 journals are collected in the total link strength. The 5 journals, as follows: *Biomaterials* (total citations = 1229 times), *American Journal of Sports Medicine* (total citations = 1180 times), *Osteoarthritis and Cartilage* (total citations = 896 times), *Journal of Orthopedic Research* (total citations = 645 times), and *Arthroscopy* (total citations = 642 times), owned best total link strength, as shown in Table 5.

**TABLE 4:** The top 10 most productive journals related to stem cells for meniscal regeneration.

Rank	Journal	Article counts	Percentage
1	Stem Cell Research Therapy	17	4.91
2	American Journal of Sports Medicine	15	4.34
3	Journal of Orthopaedic Research	13	3.76
4	Acta Biomaterialia	12	3.47
5	Osteoarthritis and Cartilage	9	2.60
6	Arthroscopy the Journal of Arthroscopic and Related Surgery	8	2.31
7	International Journal of Molecular Sciences	8	2.31
8	Journal of Tissue Engineering and Regenerative Medicine	8	2.31
7	Stem Cells International	7	2.02
10	Tissue Engineering Part C Methods	7	2.02

**TABLE 5:** The top 10 cocited journals related to stem cells for meniscal regeneration.

Rank	Cited journal	Citations	JCR (2021)	IF
1	Biomaterials	1229	Q1	15.30
2	American Journal of Sports Medicine	1180	Q1	7.01
3	Osteoarthritis and Cartilage	896	Q1	7.51
4	Journal of Orthopedic Research	645	Q2	3.10
5	Arthroscopy	642	Q1	5.97
6	Tissue Engineering Part A	596	Q2	4.08
7	Journal of Bone Joint Surgery-American	502	Q1	6.56
8	Acta Biomaterialia	501	Q1	10.63
9	Arthritis Rheumatology	484	Q1	15.48
10	Clinical Orthopedic and Related Research	421	Q1	4.76

A list of research orientations is summarized using VOSviewer in Table 6. As a matter of fact, the most prevalent research fields are cell biology, orthopedics, engineering, materials science and research experimental medicine. The main research orientation points out the current research focus and potential.

**TABLE 6:** The top 10 well-represented research areas.

Rank	Research areas	Records	Percentage
1	Cell Biology	103	29.77
2	Orthopedics	87	25.15
3	Engineering	75	21.68
4	Materials Science	67	19.36
5	Research Experimental Medicine	47	13.58
6	Sport Sciences	38	10.98
7	Biotechnology Applied Microbiology	36	10.41
8	Science Technology Other Topics	26	7.51
9	Surgery	23	6.65
10	Rheumatology	22	6.36

### 3.8 Analysis of references and funds

**FIGURE 10:**
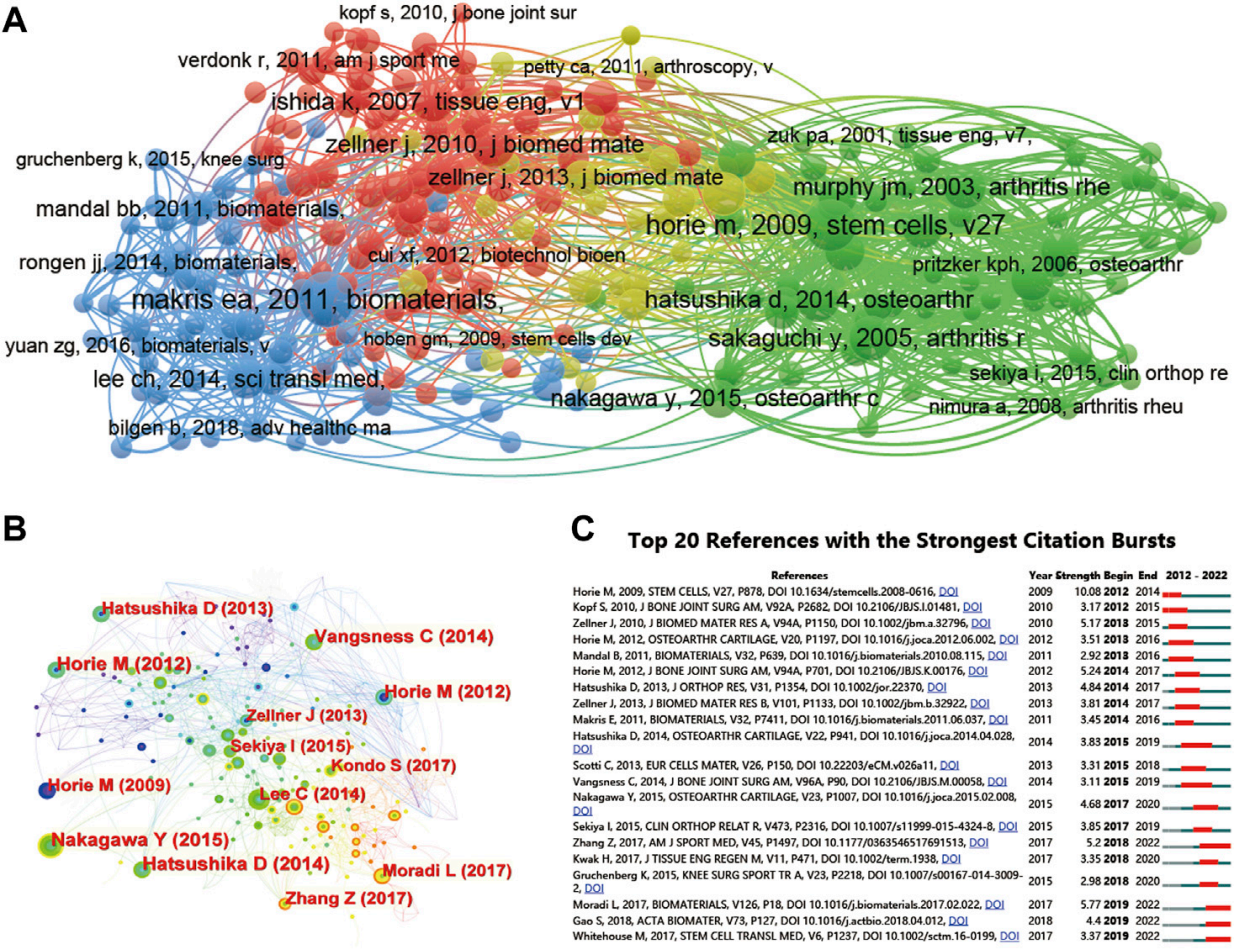
Mapping of references in studies on stem cells for meniscal regeneration. **(A)** Network map of publishing document analysis based on Vosviewer. **(B)** Network map of cocitation analysis of references with more than 50 citations based on CiteSpace. **(C)** Top 20 references with the strongest citation bursts of publications related to stem cells for meniscal regeneration.

Moreover, to show the most influential literature, cocited references were analysed by VOSviewer (Figure 10A). In this research, the publications were identified by CiteSpace and are presented in Figure 10B, as the 20 influential articles with the strongest citation bursts and the cocitation relationship between each article is presented in the network map. Moreover, cocited references were analysed by CiteSpace (Table 7), showing the top 5 most influential studies. The article titled “The knee meniscus: structure‒function, pathophysiology, current repair techniques, and prospects for regeneration”, published in 2011, was cited 85 times. In addition, more details of related articles are displayed in Figure 10C, including the citation burst for the duration of references. The paper by Horie M published in 2009 has the strongest reference citation burst, lasting from 2012 to 2014.

**TABLE 7:** The top 5 cocitation analyses of cited references on stem cells for meniscal regeneration.

Rank	Title	First author	Journal	IF	Publication year	Total citations
1	The knee meniscus: structure-function, pathophysiology, current repair techniques, and prospects for regeneration	Makris, Eleftherios A	Biomaterials	15.304	2011	85
2	Intra-articular Injected synovial stem cells differentiate into meniscal cells directly and promote meniscal regeneration without mobilization to distant organs in rat massive meniscal defect	Horie, Masafumi	Stem cells	5.845	2009	75
3	Comparison of human stem cells derived from various mesenchymal tissues: superiority of synovium as a cell source	Sakaguchi, Yusuke	Arthritis and rheumatism	—	2005	56
4	Role of mesenchymal stem cells in tissue engineering of meniscus	Zellner, Johannes	Journal of biomedical materials research. Part A	4.854	2010	52
5	Repetitive allogeneic intraarticular injections of synovial mesenchymal stem cells promote meniscus regeneration in a porcine massive meniscus defect model	Hatsushika, D	Osteoarthritis and cartilage	7.507	2014	51

A list of the funds for stem cells in meniscal regeneration is summarized in Table 8. According to Table 8, the National Natural Science Foundation of China supports the most articles with 60 articles, followed by the National Institutes of Health with 54 articles and the United States Department of Health Human Services with 54 articles. Interestingly, the first three funds that support most articles are funds from the United States and China, which is consistent with previous analyses of countries.

**TABLE 8:** The top 10 funds related to stem cells for meniscal regeneration.

Rank	Journal	Article counts	Percentage
1	National Natural Science Foundation of China Nsfc	60	17.34
2	National Institutes of Health Nih Usa	54	15.61
3	United States Department of Health Human Services	54	15.61
4	Nih National Institute of Arthritis Musculoskeletal Skin Diseases Niams	24	6.94
5	Ministry of Education Culture Sports Science and Technology Japan Mext	23	6.65
6	Japan Society for The Promotion of Science	21	6.07
7	Grants in Aid for Scientific Research Kakenhi	17	4.91
8	National High Technology Research and Development Program of China	16	4.62
7	Japan Agency For Medical Research and Development Amed	14	4.05
10	National Key R D Program of China	14	4.05

### 3.9 Analysis of keywords and hotspots

**FIGURE 11:**
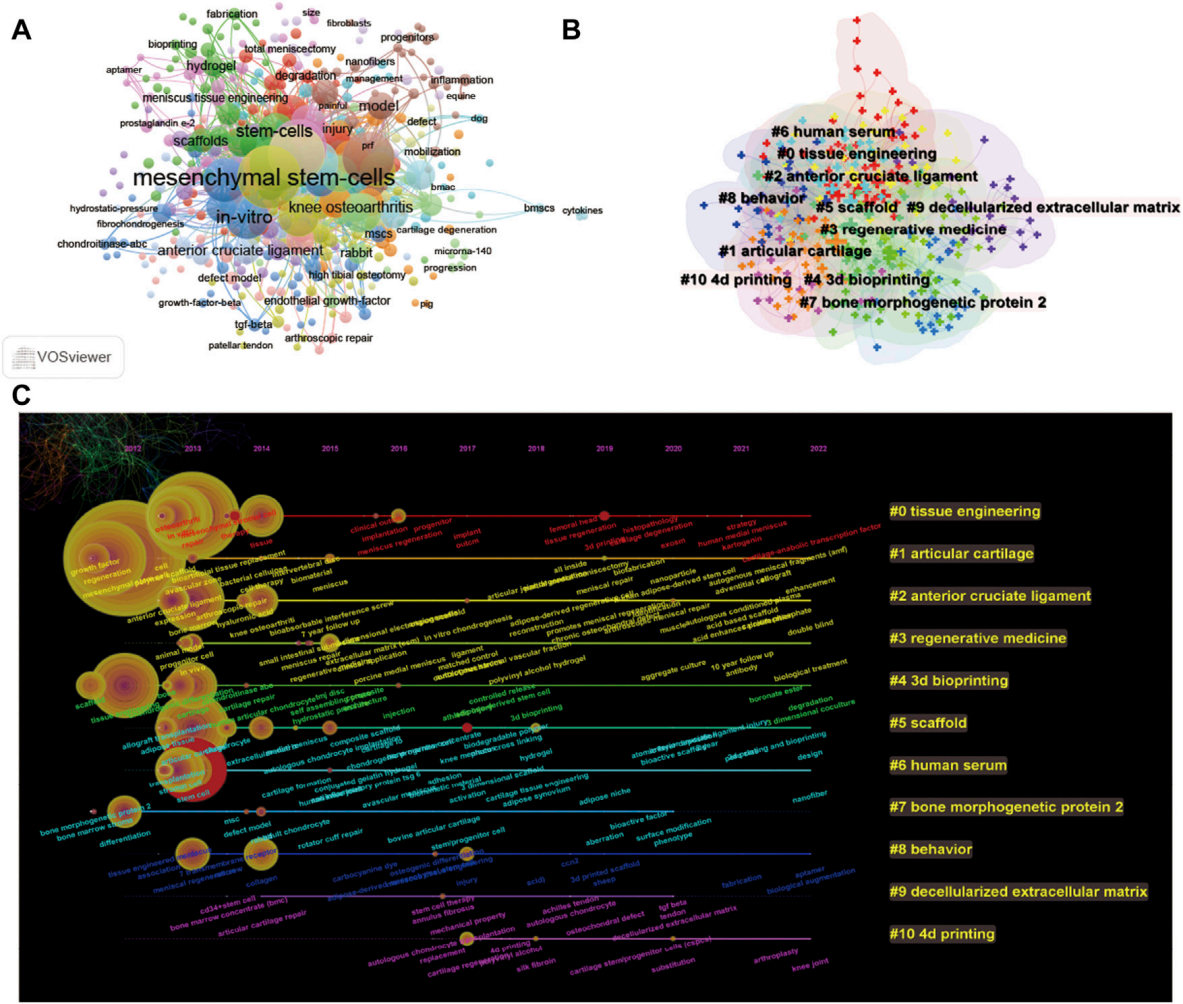
Mapping of keywords in studies on stem cells for meniscal regeneration. **(A)** Network visualization of keywords based on Vosviewer. **(B)** Keyword clustering visualization from 2012 to 2022. **(C)** Keyword timeline visualization from 2012 to 2022.

Using CiteSpace’s algorithm, the burst of keywords based on burst detection was also analysed and visualized. The top 25 keywords with the highest frequency of occurrence are shown in Figure 11A. It has been reported that the keyword with the most frequent citations is “mesenchymal stem cell”, which means it is probably a hotspot for using mesenchymal stem cells for meniscal regeneration. Furthermore, “scaffold”, “knee osteoarthritis”, and “*in vitro*” also occur frequently as the key words in the period of 2012–2022 about stem cells for meniscus regeneration. The three key words mentioned above demonstrated the attention of researchers from three perspectives: “auxiliary materials”, “experiment method”, and “disease association”.

A network map was also built to visualize keyword clusters (Figure 11B), and each node represents a hot key word using Vosviewer. Accordingly, the top ten keywords in order are #1tissue engineering, #2 articular cartilage, #3 anterior cruciate ligament, #4 regenerative medicine, #5 scaffold, #6 human serum, #7 bone morphogenetic protein 2, #8 behavior, #9 decellularized extracellular matrix, and #10 4D printing. In terms of the information, we analyse the key word transform during 2012–2022 and visualize the timeline of the related key words. As Figure 11C suggests, the frequency of key words displays a large difference with time, which undoubtedly indicates a change in research direction.

## 4 Discussion

In the past decade, enormous efforts have been made in stem cell therapy for meniscal regeneration (Pillai et al., 2018; Zhang et al., 2018), and considerable advances have been achieved in both clinical and preclinical experiments for meniscal disease. This study provided a bibliometric and visualization analysis of stem cells for meniscal regeneration from 2012 to 2022. With the development of bibliometric software, bibliometric analysis is now broadly pursued. It can favor beginners understand the development process and trends of a specific field intuitively and systematically. Additionally, it is also beneficial to find new research hotspots and milestone achievements.

### 4.1 Trend overview of the development of stem cell treatment for meniscal regeneration

As suggested in the visualized figures, there is a considerable increase in the number of publications from 1 October 2012 to 1 October 2022 despite a slight decrease in 2016. Meanwhile, the relative research interest has also increased over the past few years, indicating that the popularity of this field is also experiencing a boom.

When it comes to national contributions, in this analysis, roughly 31 countries published papers on stem cell therapy for the meniscus regeneration field. In particular, the United States contributed the most papers (117) compared to China (90), Japan (58), Germany (23), and Italy (22). The main research regions are located in North America, North America and East Asia, which have both developed economies and large populations. It is widely acknowledged that the number of total citations, average citation and the H-index are significant statistic indexes for bibliometric studies. What is interesting in Figure 3 is that the United States published the most papers (117), contributed the most extensive total citations (4233), and the largest H-index (33). These indexes suggest United States has an outstanding role in the aspects of the quality and academic impact among different countries. This phenomenon perhaps resulted from the rich resources of researchers and institutions worldwide, guaranteeing the USA’s dominant position in the field of stem cell therapy for meniscal regeneration resulting the United States, an extremely productive and chief country in this field.

Interestingly, Spain ranked top 1 in terms of average citations (41.6), with Switzerland (36.9) and the United States (35.7) following. Allowing for top 10 total publications, Spain, ranking seventh in terms of the publishing number, is still making a significant progression in this field. Considering the high number of total publications, however, China shows a disappointing performance in average citations. As a matter of fact, China ranks only eighth, which means a pretty low acceptance in the global academic research field of stem cell therapy for meniscal regeneration. The incongruity between the quality and quantity of studies also indicates that China requires more influential studies.

As for the scientific institutions, Tokyo Medical Dental University ranked first (34 publications), Peking University (29 publications), and Chinese People Liberation Army General Hospital (15 publications) have contributed a lot to the research field effectively. What is interesting is that the leading top 2 institutes have contributed considerably concerning publication number, while there exists a huge difference in average citations. In detail, the average citation of Peking University is only 17.52, while that of Tokyo Medical Dental University is up to 34.19. It is contended that the prevailing role of first-group institutes guarantees one country’s academic research ranking, allowing for approximately the top 10 institutes come from the several related countries. It can be concluded that the following related studies with cooperation could make a big difference in meniscus research, benefitting researchers producing more impactful researches across disciplines.

### 4.2 Status of authors and studies

What is surprising is that Japanese and Chinese are the first-ranked authors with the most articles, instead of the United States. However, the largest fund provided in the field is the US National Institutes of Health, which is strange. In Table 9, the top-ranked authors with the most researches were scientists with longer career and research time. In that sense, they possibly have turned attention to advancements in stem cell therapy for meniscal regeneration ahead of time. In fact, Sekiya I, the researcher with the most publications, tried to perform intraarticular injections of MSCs to promote meniscus regeneration in a porcine massive meniscal defect model and proved the protection efficiency at the medial femoral articular cartilage before 2014 (Hatsushika et al., 2014).

**TABLE 9:** The top 10 authors with the most publications on stem cells for meniscal regeneration.

Rank	High published authors	Country	Article counts	Percentage
1	Sekiya I	Japan	31	8.96
2	Koga H	Japan	27	7.80
3	Tsuji K	Japan	22	6.36
4	Muneta T	Japan	21	6.07
5	Mizuno M	Japan	19	5.49
6	Guo QY	China	16	4.62
7	Liu SY	China	16	4.62
8	Ozeki N	Japan	15	4.34
9	Yuan ZG	China	13	3.76
10	Katano H	Japan	12	3.47

What can’t be ignored is that the collaboration analysis in Figure 6B indicated a shortage of academic collaboration and close communication among authors form different countries. It can be seen that the academic relationship basically located in the same countries and is scattered in different countries. The collaboration circle mainly consists of Chinese authors and Japanese authors separately, however, with almost no intersection. Therefore, authors may enhance their collaboration in this field jointly.

For cocitation frequency, as shown in Table 10, Horie, Zellner and Arnoczky are with the highest cocitation frequency. It undoubtedly reflected the international impact and recognition of these researchers in this field. Zellner, using a combination of mesenchymal stem cells and a hyaluronan collagen-based scaffold, attempted to repair meniscal tears in the avascular zone of meniscus (Zellner et al., 2013). In addition to the authors’ analysis, the related journals concerning researches were further explored, analyzed and then shown in Table 4. The journal *Stem Cells Research Therapy*, *American Journal of Sports Medicine*, and *Journal of Orthopaedic Research* published the most papers. Meanwhile, the related journals only occupy a limited percentage, and articles are relatively scattered in different journals without aggregations. What is interesting is that more than 60 articles were published in only the top 5 journals, indicating the focus and relevance in this field. Accordingly, the listed top 10 journals might be possible choices for researchers to publish related research in the future. Furthermore, this study has also conducted cocitation analysis based on journals for investigation of the impacts of publications after analysing the total number of citations. Figure 8C shows that *Biomaterials* (IF = 15.304) made the best contributions in this field, with 1229 citations. The article published on *Biomaterials*, The knee meniscus: Structure–function, pathophysiology, current repair techniques, and prospects for regeneration (Makris et al., 2011), systematically investigated the properties of a series of scaffolds and cell types for meniscus regeneration, which produced great academic impact.

**TABLE 10:** The top 10 cocited authors on stem cells for meniscal regeneration.

Rank	High Co-cited authors	Total citations
1	Horie, M	166
2	Zellner, J	125
3	Arnoczky, Sp	111
4	Makris, Ea	104
5	Caplan, Ai	101
6	Hatsushika, D	96
7	Sekiya, I	94
8	Baker, Bm	90
9	Englund, M	84
10	Zhang, Zz	77

In addition, the top 10 research orientations are composed of biology, medicine and material engineering, which means it is a multidisciplinary field of deep intersection. More specifically, both focus and relevance of research in tissue engineering studies were reflected by the dual-map analysis.

Based on the citation analysis of documents (Figure 10A) the impact of each publication was analysed. Besides, the cocitation network analysis (Figure 10B). Table 3 shows that the most cited article is Treatment of Knee Osteoarthritis with Autologous Mesenchymal Stem Cells: A Pilot Study (Orozco et al., 2013), which may be a target for meniscus defect treatment. Another study reported a clinical double-blind controlled study using MSC for meniscectomy written by Vangsness (Vangsness et al., 2014). Among the publications, the basic research type consists of main type of publications, involving pathology, biomaterial, and preclinical research.

Notably, cocitation analysis can determine which publications have contributed most in this field. As shown in Figure 10B and Table 7, “The knee meniscus: structure‒function, pathophysiology, current repair techniques, and prospects for regeneration” (Makris et al., 2011) authored by Makris, Eleftherios et al., as the top publication, possess the highest citation frequency, which, as mentioned before, reviewed the structure, pathophysiology, repair and prospects of the meniscus. In Figure 10C, as for the strongest citation bursts, meniscus pathophysiology, diagnosis, and therapy are the aspects with most attention for the top 20 cited articles, suggesting which way hot topics exist in this research field.

### 4.3 Analysis of research hotspots

The research trends and frontiers in stem cell therapy for meniscus regeneration research could be demonstrated by the co-occurrence analysis of keywords and bursts. From Figure 11A, as the keyword with the highest citation outbreaks, “mesenchymal stem cells” shows the prevailing status of this hotspots in meniscus research. As a matter of fact, as early as 2005, Yasunori Izuta et al. attempted to utilize bone marrow-derived mesenchymal stem cells to treat meniscal tears in rats and verified a reduction in meniscus destruction in this therapy (Izuta et al., 2005). As shown in Figures 11B, C, it can be argued that the primary research clusters mainly refer to “tissue engineering”, “articular cartilage”, “anterior cruciate ligament”, “regeneration medicine”, and “bioprinting”, indicating that there is a huge similarity and connection in the whole joint cartilage and biomaterial and engineering method, all contributing greatly to meniscus regeneration.

In our study, the keyword timeline visualization network is plotted depending on the keywords in the titles/abstracts of all included publications. Figure 11C shows the 10 main research clusters, which include some more detailed secondary research keywords with their occurrence and variation over time. Not only could these keyword clusters comply with promising hotspots in the field of stem cell therapy for meniscus regeneration research, but these timelines also forecast the promising perspectives of future studies, as follows.

(I). Tissue engineering: Co-occurrence analysis of keywords identified “mesenchymal stem cell”, “regeneration”, and “implantation” as important research hotspots that deserve further attention. It is commonly known that cartilage (including the meniscus) regeneration is an important component in tissue regeneration, meaning that there is a similarity between these two fields. As a result, some advanced biomaterials or mechanisms in tissue regeneration may promote and inspire meniscal regeneration. As reviewed in this review, poly (lactic acid)-based biomaterials for orthopedic regenerative engineering suggest that the architecture, topography, and biochemical cues that influence cellular outcomes in tissue engineering can inspire research related to meniscus regeneration (Narayanan et al., 2016). Considering the specificity of the meniscus, however, implantation and regenerative effects are the main therapy and evaluation gold standards (Bansal et al., 2017). The blood supply to the meniscus comes from branches of the internal and external knee arteries, which form a network of blood vessels within the joint capsule. These arterial networks from the joint capsule and synovium only provide blood supply to 30% of the fibers around the meniscus, which is called the red zone under arthroscopy, and can be repaired after injury (Wang et al., 2022). In contrast, the central part has no blood supply, so it is called the white area. It relies on the penetration of synovial fluid for nutrition, so it lacks the ability to repair and regenerate after injury. Even worse, repetitive movement can easily induce inflammation. Therefore, exogenous implants, including MSCs, generally have a better therapeutic effect (Chen et al., 2018). Overall, both similarities and differences exist in meniscal regeneration and the keyword cluster “tissue engineering”, which may bring about benefits for future studies.
(II). Articular cartilage: Due to the low vascularization and slow metabolism, the tissue in the joint faces a similar conundrum in regeneration medicine, including the meniscus, articular cartilage and anterior cruciate ligament (Kwon et al., 2019). In fact, after injections of allogeneic synovial MSCs, articular cartilage, subchondral bone and meniscus at the medial femoral condyle were also significantly more preserved (Hatsushika et al., 2014), indicating a strong similarity and interaction in the whole joint (Chen et al., 2017). In these three clusters, “extracellular matrix *in vitro* chondrogenesis” and “hydrogel”occur with high frequency as keywords, suggesting that research on the interaction of ECM and chondrocytes would probably help with field promotion, including the mechanism, material design and application for different parts of whole joint regeneration.
(III). Regeneration medicine: Most studies share the belief that clinical experiments are the most convincing evaluation of the efficacy of stem cell therapy for regeneration medicine. In this cluster, “double blind”, “matched control” and “several years follow up” make demands on the related studies in the future. As a general rule, double-blind experiments with matched groups are compulsive in a clinical trial. Deep down, it is better to design a follow-up in a clinical trial, which will absolutely broaden the influence and scope. The chief reason is that the treatment of the meniscus is a long process, and it is impossible to obtain immediate results (Andia and Maffulli, 2017). In this sense, a suitable follow-up will undoubtedly enhance comprehensiveness and reasonableness. Moreover, according to a highly cited review of surgical and tissue engineering strategies for articular cartilage and meniscus repair (Kwon et al., 2019), strategies of tissue engineering are indispensable for meniscus repair in regeneration medicine and clinical translation.

Most human clinical trials have been highly cited, suggesting most researchers pay attention on clinical trials which only contains cells. And some stem cell therapies for meniscal regeneration based on animal model including rats, pigs and horse involve both cells and biomaterial. The two types of researches respectively represent feasibility and innovation.

(IV). 3D bioprinting

One important technology for meniscus treatment is 3D bioprinting. It is generally agreed that the repaired meniscus can function well based on the assumption that the meniscus has a correct shape and good topography that allows a stable load force transfer between the tibia and femur in the joint (Hao et al., 2021). Moreover, whether on the size or the angle, there always exists a slight difference in the patients’ menisci (Deng et al., 2021). Faced with this problem, 3D bioprinting can be used to manufacture artificial menisci containing MSCs with any shape and angle, which will certainly facilitate the individualized treatment of patients (Filardo et al., 2019). There is much potential about the association with 3D bioprinting, which needs more updated and fantastic studies.

(V). Scaffold:

Both chondrocytes and mesenchymal stem cells tend to grow in scaffolds with good activity, a higher surface area and abundant adhesion sites. Accordingly, microspheres and porous scaffolds are usually utilized in stem cell therapy for meniscal regeneration (Itose et al., 2022). In addition, it has been reported that the stiffness and viscoelasticity of the matrix significantly induce the differentiation of MSCs into chondrogenic cells, osteogenic cells and adipogenic cells (Duarte Campos et al., 2015). In the meniscus, the red zone and the white zone result in different demands for scaffolds for regeneration. It has been reported that the optimal strategy for avascular zone regeneration is a combination of scaffolds and MSCs, which is even better than growth factors alone (Zellner et al., 2010). As a result, it is likely to control stem cell differentiation to focus on different therapeutic effects by tailoring scaffolds with different physical and chemical properties.

In terms of cell types, various stem cells have been used to treat meniscal disease, including BM-MSCs (bone marrow-derived mesenchymal stem cells), AD-MSCs (adipose tissue-derived mesenchymal stem cells), US-MSCs (umbilical cord mesenchymal stem cells), ESCs (embryonic stem cells), and iPSCs (Induced pluripotent stem cells), which own different mechanism and treatment effects. BM-MSCs is the most commonly used cell types in meniscal regeneration due to its low inflammation reaction, good biocompatibility and stable therapeutic effects while AD-MSCs are easily accessible *via* minimally invasive procedures.

### 4.4 Future research trends

Stem cell therapy aims to alleviate local symptoms, help regenerate the meniscus and promote the function of patients’ movement. Accordingly, there are many urgent problems to be solved, although some clinical trials are in progress around the world. Not only safety, side effects, and long-term efficiency but also suitable cell types, ethical considerations, reasonable therapeutic courses and doses are totally unclear and unconfirmed, thus leading to serious concern and an intense debate about cell therapy for meniscal regeneration. In fact, these studies mainly focus on the possible benefits of cell therapy, the final therapeutic results and therapeutic potential, which means that the great majority of studies are not comprehensive and not specific. It is likely caused by the deficiency of the pathology and mechanism. First, meniscus defects are commonly accompanied by chronic physiological changes and intense inflammation. Meanwhile, the normal formation of the meniscus during development relates to a rich variety of cells. Unfortunately, most researchers are vague about the disease process due to complexity, which undoubtedly requires further efforts, especially at the molecular and cellular levels. In addition, the main therapeutic mechanism is uncertain, despite several hypotheses proposed and good therapeutic effects. In a highly cited review (Hofer and Tuan, 2016), it was summarized that recent mechanistic insights into trophic activities focus on ultimate regulation by nitric oxide, nuclear factor-kB, and indoleamine, among other signaling pathways, to alleviate patients’ pain and help tissue regeneration.

For instance, it is to be determined whether MSCs function mainly through differentiation into chondrocyte or paracrine forms to help meniscal regeneration. Further studies concerning pathology and mechanism will surely have a greater impact, benefiting the improvement of cell types, therapeutic course and dose, treatment method and adverse effects.

### 4.5 Limitation

There are still some limitations to be discussed: (1) Only research and review articles in English were extracted, and as a matter of fact, the articles written in non-English language or non-research/review articles were excluded in this study, which perhaps bring about some omissions. (2) Due to the limitation of our bibliometric software, publication bias may occur when some databases have not been included. In that sense, more data sources and powerful software are supposed to be included in future research. (3) Prediction bias in hotspots would result from the neglect of temporal data since the keywords with a timeline haven’t been visualized. (4) Allowing for new studies to be updated daily, some influential newly published studies would inevitably be neglected. (5) Encountered problems were resolved by consulting with experts to reach the final consensus because the data selection was performed by two authors. (6) Other public databases including Scopus could be involved to guarantee the analysis more comprehensive and representative, which is widely utilized in many similar bibliometrics and visual analysis. (7) All data were cleaned and analysed individually by the coauthors. Problems in this research was generally solved by the repeated discussion by the two authors. However, this method cannot guarantee absolute accuracy and may cause some mistakes.

## 5 Conclusion

To conclude, this study is the first bibliometric and visualization analysis to scientifically and systematically analyse global stem cell therapy for meniscal regeneration research trends over the last decade. This study systematically summarized and analyzed the global publication trends and supported researchers to identify the essential authors, journals and institutions in this field. Furthermore, the keyword and cocitation clustering analysis also inspire researchers to choose research frontiers mainly in five directions: “Tissue engineering”, “Articular cartilage”, “Regeneration medicine”, “3D-bioprinting”, and “Scaffold”. Further cooperation among authors, institutions, and countries is waiting to see, which points out the research potential and perhaps facilitates the development of stem cell therapy for meniscus regeneration research.

## Data Availability

The original contributions presented in the study are included in the article/Supplementary Material, further inquiries can be directed to the corresponding authors.
